# Viral based vaccine TG4010 induces broadening of specific immune response and improves outcome in advanced NSCLC

**DOI:** 10.1186/s40425-017-0274-x

**Published:** 2017-09-19

**Authors:** Caroline Tosch, Bérangère Bastien, Luc Barraud, Benoit Grellier, Virginie Nourtier, Murielle Gantzer, Jean Marc Limacher, Eric Quemeneur, Kaïdre Bendjama, Xavier Préville

**Affiliations:** 1 Transgene SA, 400 Bld Gonthier d’Andernach, Parc d’Innovation, CS80166, 67405 Illkirch Graffenstaden, Cedex France; 2 Current address: Department of Medical Oncology and Clinical Hematology, Louis Pasteur Hospital, 39 Av de la Liberté, 68000 Colmar, France; 3 Current address: Amoneta Diagnostics, 17 rue du Fort, 68330 Huningue, France

**Keywords:** Cancer vaccine, Viral vaccine, Tumor associated antigen, Lung cancer, T cell response

## Abstract

**Background:**

Advanced non-small cell lung cancer patients receiving TG4010, a therapeutic viral vaccine encoding human Mucin 1 and interleukin-2 in addition to standard chemotherapy, displayed longer overall survival in comparison to that of patients treated with standard chemotherapy alone. Our study intended to establish the association between overall survival and vaccine-induced T cell responses against tumor associated antigens (TAA) targeted by the vaccine.

**Method:**

The TIME trial was a placebo-controlled, randomized phase II study aimed at assessing efficacy of TG4010 with chemotherapy in NSCLC. 78 patients from the TIME study carrying the HLA-A02*01 haplotype were analyzed using combinatorial encoding of MHC multimers to detect low frequencies of cellular immune responses to TG4010 and other unrelated TAA.

**Results:**

We report that improvement of survival under TG4010 treatment correlated with development of T cell responses against MUC1. Interestingly, responses against MUC1 were associated with broadening of CD8 responses against non-targeted TAA, thus demonstrating induction of epitope spreading.

**Conclusion:**

Our results support the causality of specific T-cell response in improved survival in NSCLC. Additionally, vaccine induced epitope spreading to other TAA participates to the enrichment of the diversity of the anti-tumor response. Hence, TG4010 appears as a useful therapeutic option to maximize response rate and clinical benefit in association with other targeted immuno-modulators.

**Trial registration:**

Registered on ClinicalTrials.gov under identifier NCT01383148 on June 23rd, 2011.

**Electronic supplementary material:**

The online version of this article (doi:10.1186/s40425-017-0274-x) contains supplementary material, which is available to authorized users.

## Background

The success of immune checkpoint blockers in indications of high medical need i.e. unresectable melanoma or advanced lung cancer [1] has renewed the long standing interest for immune based therapies in clinical oncology, including therapeutic cancer vaccines.

The rationale behind therapeutic vaccination is that specific cellular response against tumor antigens would translate into an excellent safety and tolerability profiles, along with a sustainable effect likely to prevent disease progression, as well as relapse. Accordingly, numerous studies have reported a clear patient benefit in various cancer types, including aggressive tumors such as non-small cell lung cancer (NSCLC) [2]. However, clinical results are seldom supported by mechanistic evidence underlying the specificity and diversity of CD8+ T-cell reactivity.

In this study, we provide first data reporting the link between the development of a specific immune response and clinical benefit for a viral-based immunotherapeutic in advanced NSCLC. TG4010 is a therapeutic cancer vaccine based on a modified vaccinia Ankara strain (MVA) encoding for the full-length cancer antigen Mucin 1 (MUC1) and human IL-2. Administration of TG4010 in combination with chemotherapy (CT) has resulted in improved clinical outcomes in several clinical studies over the standard chemotherapy regimen [3, 4]. We used combinatorial two color encoding of MHC multimers [5] for the parallel detection of T-cell epitopes from TG4010 and other antigens reported to be prevalent in lung cancer [6]. Interactions between the vaccine and the development of T cell responses against other neo-antigens was also evaluated to evaluate the possible development of further anti-tumor response through epitope spreading.

## Methods

### Patients and study design

The TIME trial (NCT00415818) is a double-blind, placebo-controlled, randomized phase 2b/3 clinical study aimed at assessing the combination of TG4010 with first-line chemotherapy in advanced NSCLC. Inclusion criteria included age > 18 years, histology-confirmed diagnosis of previously untreated stage IV NSCLC, expression of MUC1 in at least 50% of tumor cells, Eastern Cooperative Oncology Group performance status of 0 or 1, and adequate hepatic, renal, and hematologic function. Patients were randomly allocated to receive subcutaneous injections of either 108 plaque-forming units of TG4010 or placebo, from the beginning of chemotherapy every week for 6 weeks and then every 3 weeks up to progression (Additional file 1: Figure S1). Chemotherapy regimen were chosen by the investigator: paclitaxel and carboplatin, pemetrexed and cisplatin or gemcitabine and cisplatin. Bevacizumab and Erlotinib were allowed as maintenance therapy. The trial included 222 patients (TG4010 and chemotherapy: 111 patients [50%]; placebo and chemotherapy: 111 patients [50%]).

### Monitoring of T cell responses by combinatorial encoding of MHC multimers and validation of the method

Patients enrolled in this trial were sampled 10 mL of blood at different time points along the trial (Additional file 1: Figure S1). Samples were shipped at ambient temperature 15–25 °C in a single use shipping container validated accordingly to principle set forth in the U.S. Pharmacopeia. PBMC were extracted, frozen and stored in liquid nitrogen within 24 h after collection. To recover enough cells for the immunomonitoring, samples collected at baseline and 6 h following the first treatment were pooled to evaluate the T cell response to various antigens before treatment (Additional file 1: Figure S1). Similarly, samples collected from the first day of the third treatment cycle and thereon were pooled to measure the T cell response to the same pool of various antigens after treatment (Additional file 1: Figure S1). Frozen vials were thawed in a 37 °C water bath under agitation and then transferred to complete RPMI medium (Sigma-Aldrich, R0883) containing 10 mL of 10% FCS (PAA Laboratories, A512110908312016), 0.01 g/L gentamycin (Schering Plough, U570036), 50 U/mL Benzonase nuclease (Merck, 1,016,970,001), 10 mM/L L-glutamine (Sigma-Aldrich, G5792). PBMCs were washed once again in 10 mL of complete RPMI medium before being resuspended in 2 mL PBS 2% FCS (FACS buffer) and counted manually by Trypan blue exclusion to also determine viability. To standardize the quality of the tested samples, pooling of these various time point was conditional on the viability of the latter. The acceptance criteria for cell viability was set at 80% with a median stability of 86% for pre-treatment samples and 85% for post-treatment samples (Additional file 1: Table S1). PBMCs were then enriched in CD8+ T cells using CD8+ T Cell Isolation Kit (Miltenyi Biotech, 130–096-495) before staining with 0.5 μg of various tetramers (TC Metrix, Switzerland) for 15 min at 37 °C. Tubes were then transferred on ice and cells were further incubated for 30 min with 4 μL of 40-fold dilution of near IR live dead (Invitrogen, L10119) and 1 μL of AF700 mouse anti-human CD8a (clone HIT8a, Biolegend 300,920). After washing in FACS buffer, cells were acquired on a Becton Dickinson ARIA III cell sorter equipped with 4 lasers and 16 detectors. For each sample, all available cells were acquired. Flow cytometry files were analyzed with the Kaluza software (Beckman Coulter). The strategy of CD8+ T cells selection is described in Additional file 1: Figure S2A. Tetramer stained cells were gated as shown in a representative example in Additional file 1: Figure S2B and then Boolean gating was applied as described previously [5] to monitor T cell responses to MUC1, MVA, other tumor associated antigens described in advanced non-small cell lung cancer [6] as well as predicted neoantigens from somatic mutations [7, 8] by two-color combinatorial detection of antigen specific T cells. Flu and CMV epitopes were added as controls. Operators during sample processing and flow cytometry data analysis were blinded to treatment arm.

Prior to clinical sample analysis, this method was validated (Additional file 2: Validation method) with blood samples from healthy donors to determine the limit of blank and the limit of detection for each MHC multimer. This was based on the assumption that healthy donors have very low frequencies of circulating specific T cells against the antigens tested in this study with the exception of positive controls (hCMV and Flu). Hence, for any of the TIME trial sample, a response was considered positive as being below the limit of detection (LOD) prior to treatment and above LOD post treatment. For those patients who displayed a response to a given epitope that was above the corresponding LOD prior treatment, positivity of the response to treatment was determined by specific T CD8+ frequency increase of at least two times the corresponding standard deviation.

### Statistical analysis

Log-rank Mantel-Cox tests were used to compare survival between different groups. Hazard Ratios (HR) and corresponding 95% Confidence Interval (95% CI) were estimated using a Cox regression model. For comparisons on the number of responses between the two groups of response to MUC1, non-parametric Mann-Whitney U tests were used given the limited number of data. Prism (GraphPad Software Inc., v5) was used for graphical representation of data, and both Prism and SAS (SAS Institute Inc., Cary, NC, USA) were used for statistical testing. P values <0.05 were considered statistically significant. Differences on continuous clinical and demographic baseline parameters between subgroups of patients were tested using the non-parametric Wilcoxon-Mann-Whitney test and Fisher exact test was used for categorical parameters.

## Results

### Safety and efficacy of TG4010

**Fig. 1:**
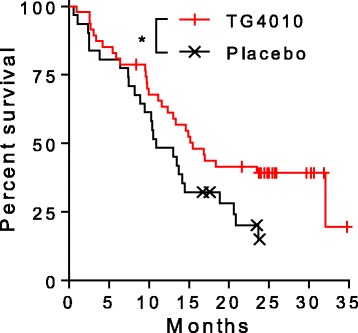
Kaplan-Meier plot of survival in HLA-A02*01 patients enrolled in the TIME study in the TG4010 (*n* = 47; red line) and placebo arms (*n* = 31; black line). Median survival of patients is 15.5 months in the TG4010 arm and 10.9 months in the placebo arm (HR = 0.58) (*: *p* < 0.05, Log-Rank test)

No grade 3–4 adverse events nor serious adverse events were considered related to TG4010 [9]. In the TIME trial, median progression-free survival (PFS) was 5.1 months and 5.9 months respectively in the placebo and TG4010 arm; overall survival (OS) was 10.6 and 12.7 month in the placebo and TG4010 arm respectively [9, 10]. When restricting the analysis to HLA-A02*01 patients, median OS was 10.9 months in the Placebo arm and 15.5 months in the TG4010 arm (HR 0.58) (Fig. 1). Samples from these HLA-A02*01 patients were analyzed here to characterize antigen specific cellular immune responses.

### Monitoring of CD8 T-cell immune response against TG4010 antigens

T-cell responses of 78 patients (47 and 31 in the TG4010 arm and placebo arm, respectively) of the TIME study carrying the HLA-A02*01 haplotype were analyzed using combinatorial encoding of MHC multimers [5, 11]. This approach allowed the multiplexed detection of CD8+ T cell response against HLA-A02*01 restricted epitopes of MUC1, of the viral vector MVA, and of 15 NSCLC-associated antigens [6–8] (Additional file 1: Table S2). Following validation of the method, including the determination of analytical sensitivity (Additional file 2: Method Validation), assessments were performed on peripheral blood mononuclear cells collected and pooled for analysis of T cell response before and after treatment (Additional file 1: Figure S1). The general gating strategy is shown (Additional file 1: Figure S2A and B) and representative examples of the two-color combinatorial measurement are shown for MUC1- (Additional file 1: Figure S3A and Figure S4A), MVA- (Additional file 1: Figure S3B and Figure S4B), tumor associated antigens RHAMM R3- and AURA B1- (Additional file 1: Figure S3C), MAGE A3, PRAME P3 (Additional file 1: Figure S4C) as well as CMV- and Flu- (Additional file 1: Figure S3D, Additional file 1: Figure S4D) specific CD8+ T cells.

**Fig. 2:**
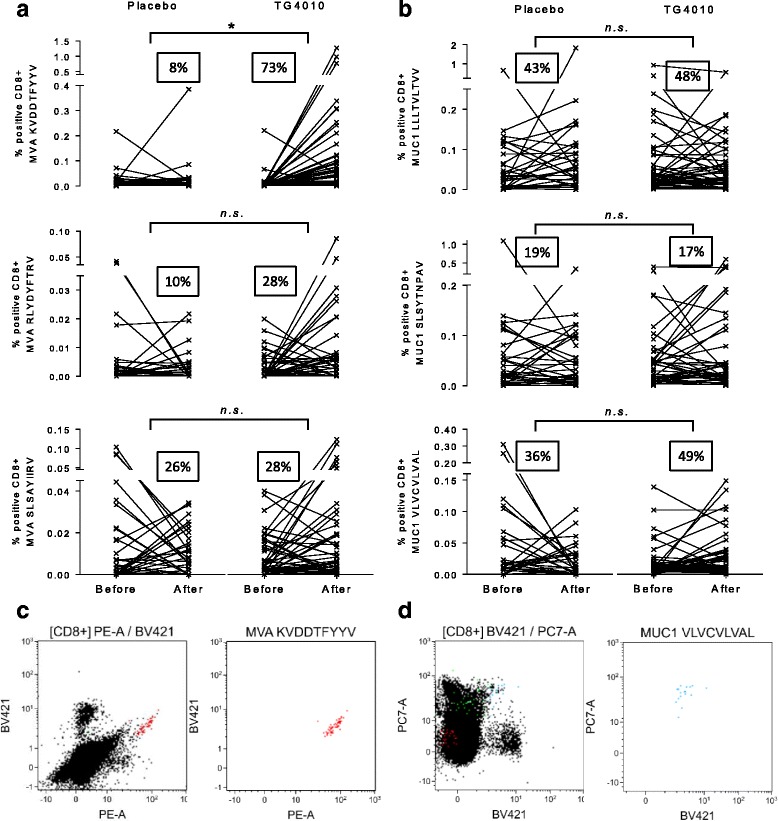
**a**. Plots of individual responses against 3 known MVA HLA-A02*01-restricted epitopes in the Placebo (*n* = 31) and TG4010 (*n* = 47) arms expressed as percentage of positive CD8+ T cells before and after treatment. The percentage of patients with an analytically significant amplification of the response during the treatment course is indicated as a squared figure in each graph. **b**. Same as in A for 3 known MUC-1 HLA-A02*01-restricted epitopes. **c**. Representative dot plot example of combinatorial encoded MHC multimer staining for one patient from the TG4010 arm for HLA-A02*01-restricted epitopes of MVA KVDDTFYYV. The x and y axis of dot plots are exponential and fluorescence is given in arbitrary units. Left dot plot displays all CD8+ events; right dot plots are restricted to the two-color positive events. **d**. Same as in C for HLA-A02*01-restricted epitope of MUC1 VLVCVLVAL. (n.s.: not significant, *: *p* < 0.05, Mann-Withney U test)

**Fig. 3:**
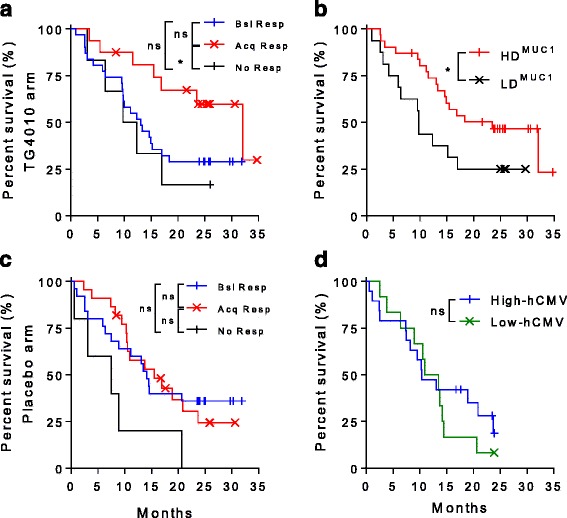
**a**. Kaplan-Meier plot of survival in patients classified per their response to MUC1 in the TG4010 arm. Patients with no MUC1-specific response (black line, No Resp. Median OS: 11 months, *n* = 6), patients who had a baseline MUC1-specific response without change upon treatment (blue line, Bsl resp., Median OS: 13 months, *n* = 24) and patient acquiring a MUC1 response during treatment (red line, Acq Resp Median OS: 32.1 months, *n* = 16). (*p < 0.05, ns: not significant, Log rank test). **b**. Kaplan-Meier plot of survival in patients in the TG4010 arm classified per the diversity of response to the MUC1 antigen of TG4010. Patients with no or only one MUC1 epitope specific response (black line, LD^MUC1^, Median OS: 9.7 months, *n* = 16). Patients with responses directed against 2 or 3 MUC1 epitopes (red line, HD^MUC1^, Median OS: 23.5 months, *n* = 31). (*: *p* < 0.05, Log rank test). **c**. Same as in A for patients of the placebo arm. Patients with no MUC1-specific response (black line, No Resp. Median OS: 7.5 months, *n* = 5), patients who had a baseline MUC1-specific response without change upon treatment (blue line, Bsl resp., Median OS: 14.1 months, *n* = 15) and patient acquiring a MUC1 response during treatment (red line, Acq Resp Median OS: 15.5 months, *n* = 11). (ns: not significant, Log rank test). **d**: Kaplan-Meier plot of survival in patients in the TG4010 arm stratified on the intensity of response against known cytomegalovirus HLA-A02*01-restricted epitope. Patients were stratified based on the response intensity and allocated to the “high” subgroup (blue line, Median OS: 10.4 months, *n* = 22) when above median or “low” group (green line, Median OS: 12.2 months, *n* = 25) when below median. (ns: not significant, Log rank test)

Consistent with repeated injections of TG4010, development of CD8+ T cell response to MVA-specific epitopes were more frequently observed in the TG4010 arm in comparison to the placebo arm (Fig. 2a). Surprisingly, frequencies of onset of responses to MUC1-specific epitopes were equivalent between both arms of the study (Fig. 2b). Representative examples of detected immune responses to MVA and MUC1 epitopes are shown in Fig. 2c and d, respectively. The development of a response against MUC1 during treatment with TG4010 was associated with an improved clinical outcome (Fig. 3a), with a median OS of 32.1 months for patients who acquired a response against at least one MUC1 epitope under TG4010 treatment versus 12.7 months in non-responders (HR 0.43 [95% CI 0.20–0.93]; p = 0.03). Furthermore, in the TG4010 arm, response against 2 or more MUC1 epitopes post-treatment was significantly associated with a longer OS (23.5 months, high diversity response; HDMUC1) as compared to patients with no response or limited to one MUC1 epitope (9.7 months, low diversity response; LDMUC1) (HR 0.48 [95% CI 0.18–0.99]; p = 0.04) (Fig. 3b). In contrast, acquisition of an immune response to MUC1 under chemotherapy alone did not result in improved OS (Fig. 3c). Demographic baseline characteristics between the patients of the different subgroups were not different (Additional file 1: Table S3 and S4) and clinical outcome was not correlated to specific response against Flu or hCMV epitopes (Fig. 3d and Additional file 1: Figure S5) indicating that the extended survival was not merely reflecting an overall better physiological status.

Results from the TIME trial [9] show that low baseline percentages of peripheral lymphocytes with CD16 + CD56 + CD69+ phenotype (TrPAL) are associated with higher clinical response to TG4010. Hence, we analyzed MUC1 response in patients with low or elevated TrPAL levels. In patient with low TrPAL levels, the population is equally segmented across LDMUC1 and HDMUC1 responders, while HDMUC1 responders are slightly underrepresented in patients with elevated TrPAL (Additional file 1: Table S5).

As indicated in the Material and Methods section, different chemotherapy regimen were used in this study. While the study is insufficiently powered to reach statistical significance, patients under cisplatin-based regimen tended to display responses with higher diversities against both MUC1 (Additional file 1: Table S6) and the viral vector (Additional file 1: Table S7) than patients under Carboplatin based regimen.

As patients included in the TIME trial had different percentages of MUC1 expression within their tumor cells, we evaluated the diversity of T cell specific response to MUC1 in subgroups of patients with different levels of MUC1 expression. There was no interaction between tumor expression of the antigen and development of an immune response (Additional file 1: Table S8).

**Fig. 4:**
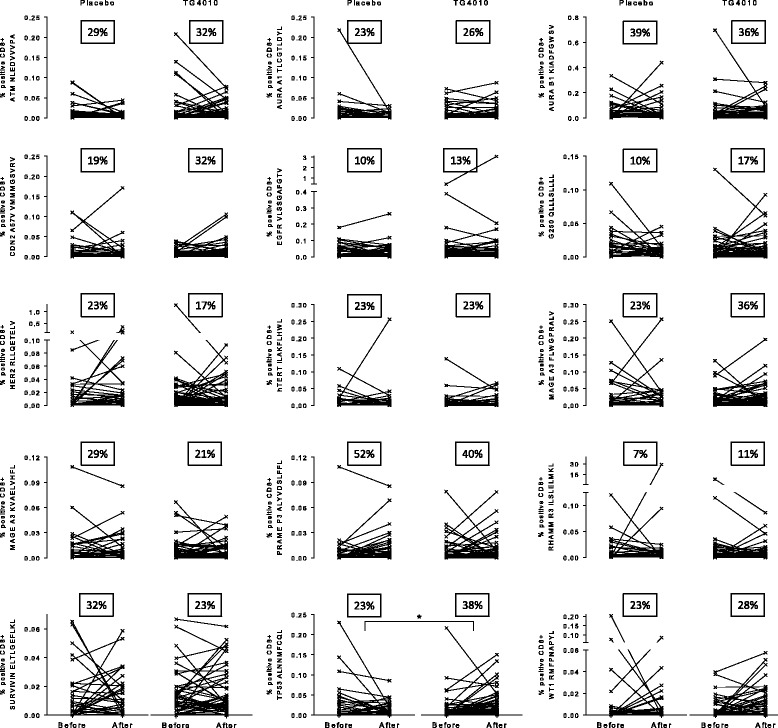
Plots of individual responses against 15 tumor associated antigens (TAA) in the Placebo (*n* = 31) and TG4010 (*n* = 47) arms expressed as percentage of positive epitope-specific CD8+ T cells before and after treatment. The percentage of patients with onset of a response or an analytically significant amplification of a preexisting response during the treatment course is indicated as a squared figure in each graph. (changes for each individual epitopes were not significant unless otherwise stated, *: *p* < 0.05, non parametric Mann-Whitney test)

**Fig. 5:**
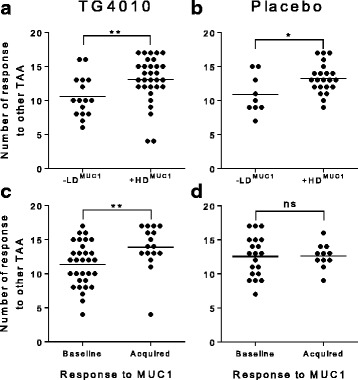
Spreading of immune response to other TAA. **a**. Number of TAA-specific responses in the TG4010 arm with patient classified per the diversity of MUC1-specific response (LD^MUC1^ (*n* = 16) vs HD^MUC1^ (*n* = 31)). Dots are representative of individual patients; the horizontal bar represents the average number of response **b**. Same as in A for patients of the placebo arm (LD^MUC1^ (*n* = 9) vs HD^MUC1^ (*n* = 22)). **c** Number of TAA-specific responses in the TG4010 arm with patients classified per the acquisition or not of MUC1-specific responses during treatment (Subjects with unchanged baseline MUC1 response without change upon treatment (*n* = 28) vs. Subjects with acquisition of a *de novo* response (*n* = 16)). **d**: Same as in C for patients of the placebo arm (Subjects with unchanged baseline MUC1 response without change upon treatment (*n* = 20) vs. Subjects with acquisition of a *de novo* response (*n* = 11)). (*: *p* < 0.05, **: *p* < 0.01, ns: not significant, non parametric Mann-Whitney test)

While not statistically significant when considering individual epitopes, amplification of TAA specific response during treatment, is more apparent in the TG4010 arm than in the placebo arm as shown by analysis of individual responses (Fig. 4). Indeed, there was a higher proportion of patients showing an amplification of response in the TG4010 arm (9 TAA out of 15) in comparison to that of the placebo arm (5 TAA out of 15). When considering the 15 lung cancer associated antigens tested, HDMUC1 patients had significantly more responses against other TAA than LDMUC1 patients after either TG4010 plus CT (p = 0.005) or CT alone (p = 0.02) (Fig. 5a and b). Noteworthy, when stratifying patients based on whether or not they acquired a response against MUC1 during treatments, patient receiving TG4010 and acquiring a MUC1 response had significantly more responses against TAA (p = 0.004) (Fig. 5c), whereas patients developing a MUC1 response under CT alone did not exhibit a higher rate of response against other TAA (Fig. 5d).

In summary, in the setting of the TIME trial, TG4010 treatment is associated with a longer overall survival for those HLA-A02*01 patients who either acquire a cellular immune response or are able to mount a diverse cellular immune response to MUC1. Spreading of the cellular immune response to other TAA is also associated with the acquisition and the diversity of the cellular response to MUC1 under TG4010 treatment.

## Discussion

To our knowledge, this work is the first report of an association between therapeutic vaccine, specific cellular immune response, broadening of this response to other TAA and improvement in clinical outcome in advanced NSCLC. Previous studies reported sporadic response immune responses in patients treated with a MUC1 vaccine [12, 13] or increases in diversity of response against epitopes that were not part of the vaccine formulation [14, 15] but these studies were limited by a low number of patients and did not allow to conclude on clinical significance of these findings. The diversity of the MUC1-specific CD8+ response was associated with a significantly longer OS. The study could not evidence any prominence for a particular MUC1 epitope but instead showed that multiplicity of the response both against MUC1 and other tumor antigenic determinants, including predicted neo-epitopes was beneficial for the patient. We had to focused on a set of 11 previously described epitope sequences [6] and further evaluated 4 predicted neo-epitopes [7, 8]. A fraction of patients developed an amplification of immune CD8+ responses against the said TAA either after receiving TG4010 combined with CT or CT alone. This fraction represented from 5% to 39% of the patient population depending on the considered TAA. Ideally, these numbers would require interpretation in light of TAA expression in patient tumor. The absence of individual information on the presence of each TAA in the study subjects constitute a limitation of our study. Despite this shortcoming, an association between response to MUC1 and TAAs could be observed. This observation is in line with the epitope spreading hypothesis suggesting that epitopes distinct from and non-cross-reactive with an inducing epitope become targets of an evolving immune response [16, 17]. Broadening of epitope recognition is likely to lead to the long-term control of tumor and to prevent negative selection of cancer cells not expressing the targeted antigen [18]. Standard chemotherapy (CT) on its own also triggered the development of immune responses against MUC1 and other tested tumor associated antigens including predicted neo-epitopes. This effect was reported for certain drug classes and is believed to result from the release of antigens upon lysis of cancer cells [19, 20], activation of cytotoxic T cells or depletion of regulatory cells by CT agents. However, in this study, acquisition of a MUC1 response under CT regimen without administration of TG4010 was not associated with an expansion of the breadth of anti-TAA responses nor with an extended OS. Hence, while the repertoire of the immune response induced by TG4010 + CT was slightly broader than that of CT alone, we believe that the difference in overall survival outcome for the patients receiving TG4010 may result from either a change of functional state of generated T cells, a modification of the composition of the tumor micro-environment or a combination of both. Our earlier preclinical work reported the ability of a subcutaneous viral based vaccination to trigger a detectable infiltration of the tumor environment by CD8+ and CD4+ lymphocytes [21]. Hence, in our case, broadening of the immune response might have synergized with CD8+ T cell enrichment of tumor sites after vaccination, consistently to what has been reported in melanoma [22]. Alternatively, it could be hypothesized that the viral nature of the vector, as well as the IL-2 encoded in TG4010, likely act as adjuvants which further assist the generation of functional T cell responses to MUC1 and other TAA [23]. For instance, it has been reported that anergic T cells in breast carcinoma have defective IL-2 secretion [24]. Furthermore, subcutaneous injection of immunotherapeutics at a distance from the tumor site has been shown to qualitatively and quantitatively influence antigen presentation with evidence that the relative distance to the tumor is positively correlated with efficacy of the induced adaptive response in both in preclinical and clinical models [25, 26]. As the vaccine induced immune response is elicited outside of the tumor immunosuppressive environment [27], it might have led to a more efficacious anti-tumor response. Although fully hypothetical at this stage of the clinical development of TG4010, it could be that functional MUC1-specific CD8+ T cells primed by TG4010, infiltrate the tumor, kill MUC1-expressing cancer cells, and thus release additional TAA that are subsequently presented to the immune system to generate more functional TAA- and neo-epitope-specific CD8+ T cells. Such an iterative broadening of anti-tumor response would fit with the cancer-immunity cycle model proposed by Chen and Mellman [28]. Future clinical monitoring of TG4010 should aim at validating this hypothesis.

Synergy between cancer vaccines and CT is a largely recognized phenomenon based on numerous preclinical and clinical observations. While the study was not intended to compare the effect of different chemotherapy regimen with respect to their interaction with the vaccine, we observed a slight tendency toward increased immune responses under cisplatin combined with pemetrexed or gemcitabine versus carboplatin and paclitaxel regimen. Accordingly, in the TIME study addition of TG4010 to CT resulted in a gain in overall response rate of 12% over placebo in patients receiving cisplatin based regimen versus 2.4% in patients receiving carboplatin plus paclitaxel. This suggests that some CT regimen may be preferable in combination with TG4010, however further studies are warranted to better characterize the interaction between immunotherapeutics and CT.

An elevated baseline peripheral ratio of lymphocytes expressing CD16, CD56 and CD69, described by Quoix et al. [9] as Triple Positive Activated Lymphocytes (TrPAL), was associated with poor clinical response and outcome under TG4010. These cells constitutes a heterogeneous cellular phenotype comprising innate immunity Natural Killer lymphocytes and, to a lesser extent, Natural Killer T cells. It is a plausible that the complex interplay between NK cells and adaptive immunity results in decreased MUC1 response. Such negative modulation was reported elsewhere through direct toxicity toward CD8+ T cells or indirectly mediated by decreased antigen presentation by dendritic cells [29]. This subgroup analysis was not sufficiently powered to reflect the observation of Quoix et al. at the level of the cellular immune response to MUC1 and other TAA in HLA-A02*01 patients, although we noticed that responders with high diversity to MUC1 were slightly under represented in those HLA-A02*01 patients with an elevated baseline peripheral ratio of TrPAL.

## Conclusion

Finally, this work demonstrates that the viral vaccine TG4010 modulates the CD8+ T cell response and that changes induced by the vaccine are associated with improvements of clinical outcome. Monitoring of specific CD8+ T cells functionality induced by TG4010 (both toward MUC1 and other TAA), should be included in future clinical trials concerning this immunotherapeutic to allow confirmation in an independent cohort, as a predictor of treatment outcome and thus, as a biomarker to support the treatment of patients with TG4010.

From the results of this trial and those of pre-clinical observations (Remy-Ziller et al., in revision), TG4010 is now pursuing its clinical development in combination with Nivolumab (NCT02823990) in second line treatment for advanced NSCLC patients and soon in combination with Nivolumab + standard chemotherapy in first line treatment for advanced NSCLC patients. Such trials as well as others with similar vaccines with immune checkpoints inhibitors that are currently ongoing, should provide precious insights on how to optimize the use of vaccines by combining it with the right immune checkpoint blocker and the right cytotoxic agent. Associated to a precise characterization of patient immune status, there is a high probability that such combined targeted interventions will harness the anti-tumor potential of the immune system and result in significant improvement in cancer care.

## Additional files

Additional file 1: **Figure S1.** Graphical description of TIME study design. Samplings for various monitoring including that of T cell response, were performed at baseline, 6 h after first injections, 15 days after the first injections, prior the third, fifth and end of treatment chemotherapy cycles. Samples pooled for analysis of T cell response before and after treatment are indicated. **Figure S2.** Gating strategy to analyze binding of various tetramers to CD8+ T cells. **Figure S3.** Representative dot plot example of combinatorial encoded MHC multimer staining for patient 0101_00019 from the TG4010 arm. **Figure S4.** Same as in Additional file 1: Figure S3 for patient 201_00001 from the TG4010 arm. **Figure S5.** Kaplan-Meier plots of survival in the TG4010 and Placebo arm stratified on response against Flu and hCMV. **Table S1.** Viability of the thawed samples used to monitor T cell response. **Table S2.** Epitopes used for the measurement of T-cell response. **Table S3.** Baseline characteristics for patients with response against 0 or 1 MUC1 epitopes (Low diversity) and 2 or 3 MUC1 epitopes (High diversity). **Table S4.** Baseline characteristics for patients with detected MUC1 response at baseline, with acquired response during treatment or with no response detected after treatment. **Table S5.** Number of patients with Low and High diversity MUC1 specific response stratified on TrPAL levels. **Table S6.** Number of patients with high and low diversity MUC1 response in each treatment arm according to the concomitant chemotherapy regimen. **Table S7.** Number of patients with high and low diversity MVA response in each treatment arm according to the concomitant chemotherapy regimen. **Table S8.** Number of patients in groups of MUC1 expression levels with low or high diversity MUC1 specific T-cell response. (DOCX 918 kb)

Additional file 2: Analytical validation summary reporting analytical performance of the combinatorial tetramer staining assay. (PDF 263 kb)

Additional file 3: List of ethics committees having reviewed and approved the study protocol. (DOCX 14 kb)

## Abbreviations

CI: Confidence interval
CMV: Cytomegalovirus
CT: Chemotherapy
HD: High diversity
HLA: Human leukocyte antigen
HR: Hazard ratio
LD: Low diversity
LOD: Limit of detection
MHC: Major histocompatibility complex
MUC1: Mucin 1
MVA: Modified vaccinia virus Ankara
NSCLC: Non small cell lung cancer
PBMC: Peripheral blood mononuclear cell
TAA: Tumor associated antigens
