# Supplementary material for: Viral based vaccine TG4010 induces broadening of specific immune response and improves outcome in advanced NSCLC
Source: J Immunother Cancer. 2017 Sep 19;5:70. doi: 10.1186/s40425-017-0274-x (PMC5604422; doi:10.1186/s40425-017-0274-x)
Supplement: Supplementary file 1 — Figure S1. Graphical description of TIME study design. Samplings for various monitoring including that of T cell response, were performed at baseline, 6 h after first injections, 15 days after the first injections, prior the third, fifth and end of treatment chemotherapy cycles. Samples pooled for analysis of T cell response before and after treatment are indicated. Figure S2. Gating strategy to analyze binding of various tetramers to CD8+ T cells. Figure S3. Representative dot plot example of combinatorial encoded MHC multimer staining for patient 0101_00019 from the TG4010 arm. Figure S4. Same as in Additional file 1: Figure S3 for patient 201_00001 from the TG4010 arm. Figure S5. Kaplan-Meier plots of survival in the TG4010 and Placebo arm stratified on response against Flu and hCMV. Table S1. Viability of the thawed samples used to monitor T cell response. Table S2. Epitopes used for the measurement of T-cell response. Table S3. Baseline characteristics for patients with response against 0 or 1 MUC1 epitopes (Low diversity) and 2 or 3 MUC1 epitopes (High diversity). Table S4. Baseline characteristics for patients with detected MUC1 response at baseline, with acquired response during treatment or with no response detected after treatment. Table S5. Number of patients with Low and High diversity MUC1 specific response stratified on TrPAL levels. Table S6. Number of patients with high and low diversity MUC1 response in each treatment arm according to the concomitant chemotherapy regimen. Table S7. Number of patients with high and low diversity MVA response in each treatment arm according to the concomitant chemotherapy regimen. Table S8. Number of patients in groups of MUC1 expression levels with low or high diversity MUC1 specific T-cell response. (DOCX 918 kb) [file 40425_2017_274_MOESM1_ESM.docx]

**Supplementary Material:**

Supplementary Fig. 1: Graphical description of TIME study design. Patients were included after histological confirmation of Stage IV non-small-cell lung cancer with immunohistological evidence of tumoral MUC1 expression on more than 50% of cells. All patients received a platin-based doublet of chemotherapy regimen (paclitaxel and carboplatin, pemetrexed and cisplatin, or gemcitabine and cisplatin, grey arrows), and given at standard doses for up to six cycles. TG4010 (at a dose of 10^8^ plaque-forming units) or the matching placebo were given by subcutaneous injections from the beginning of chemotherapy once a week for 6 weeks and then every 3 weeks up to progression (green arrows), premature discontinuation due to any reason (eg, an adverse event), or toxic effects. The placebo was the formulation buffer of TG4010. Sampling for various monitoring including that of T cell response (red arrows), were performed at baseline, 6 hours after first injections (C1D1 + 6hours), 15 days after the first injections (C1D15), prior the third, fifth and end of treatment chemotherapy cycles (C3, C5 and EoT, respectively). Samples pooled for analysis of T cell response before and after treatment are indicated.

C1

C2

C3

C4

C5

EoT

Baseline

C1D1

+6h

C1D15

C3

C5

EoT

PBMC pooled for analysis of T cell response before treatment

PBMC pooled for analysis of T cell response after treatment

Supplementary Fig. 2: Gating strategy to analyze binding of various tetramers to CD8+ T cells. A, CD8+ positive T cells were selected from living lymphocytes after doublet exclusion. B, MHC multimer positive CD8+ T cells were selected for each of the color tested (PE, APC, Brilliant Violet BV421, BV510, BV605, BV711, BV786 and PE-Cy7). Boolean gating between gates A-H generated dot plot of combinatorial encoded MHC multimer staining.


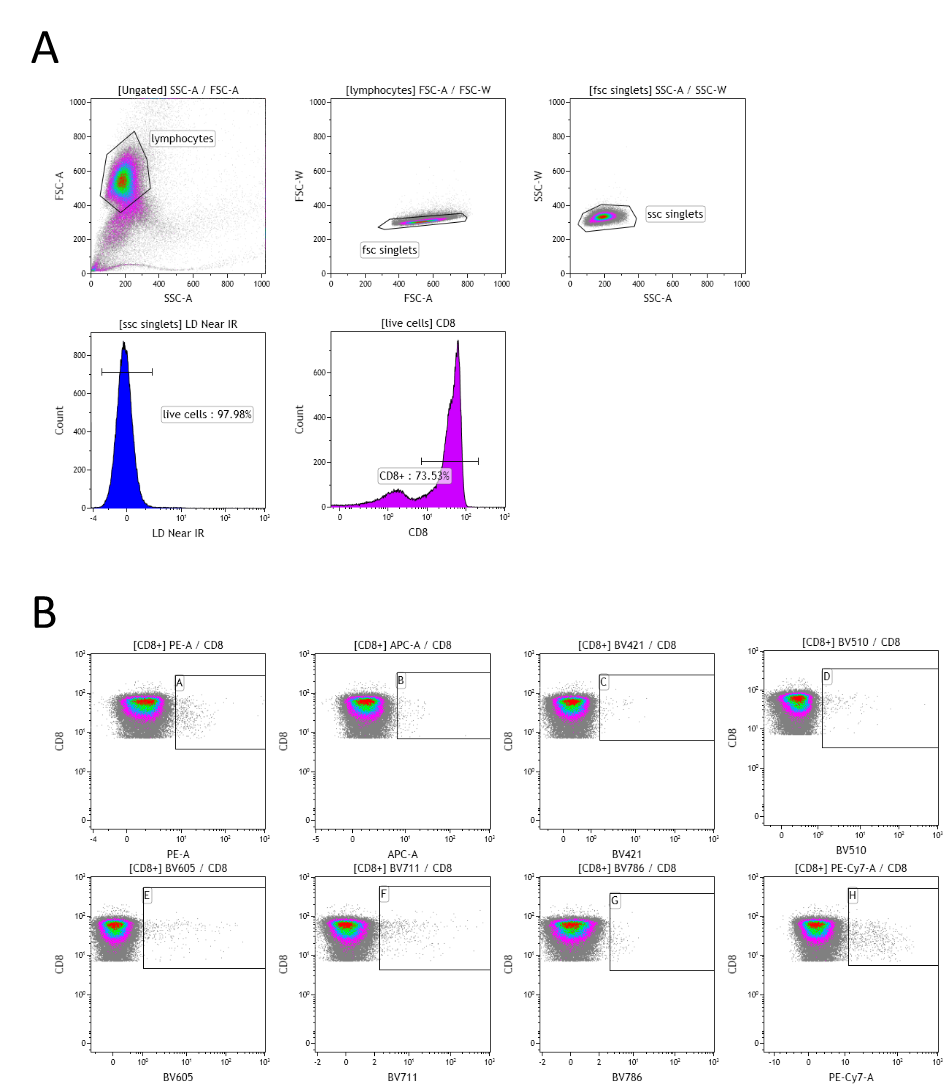


Supplementary Fig. 3: Representative dot plot example of combinatorial encoded MHC multimer staining for patient 0101_00019 from the TG4010 arm. The x and y axis of dot plots are exponential and fluorescence is given in arbitrary units. Upper plots display all CD8+ events; lower dot plots are restricted to the two-color positive events., A, HLA-A02*01-restricted epitopes of MUC1 SLSYTNPAV, LLLTVLTVV and VLVCVLVAL as indicated in lower dot plots, are shown. B, Same as in A for HLA-A02*01-restricted epitopes of MVA KVDDTFYYV, SLSAYIIRV and RLYDYFTRV, respectively. C, Same as in A for HLA-A02*01-restricted epitopes of RHAMM R3 (ILSLELMLK) and AURA B1 (KIADFGWSV). D, Same as in A for HLA-A02*01-restricted epitopes of FLU (GILGFVFTL) and hCMV (NLVPMVATV).


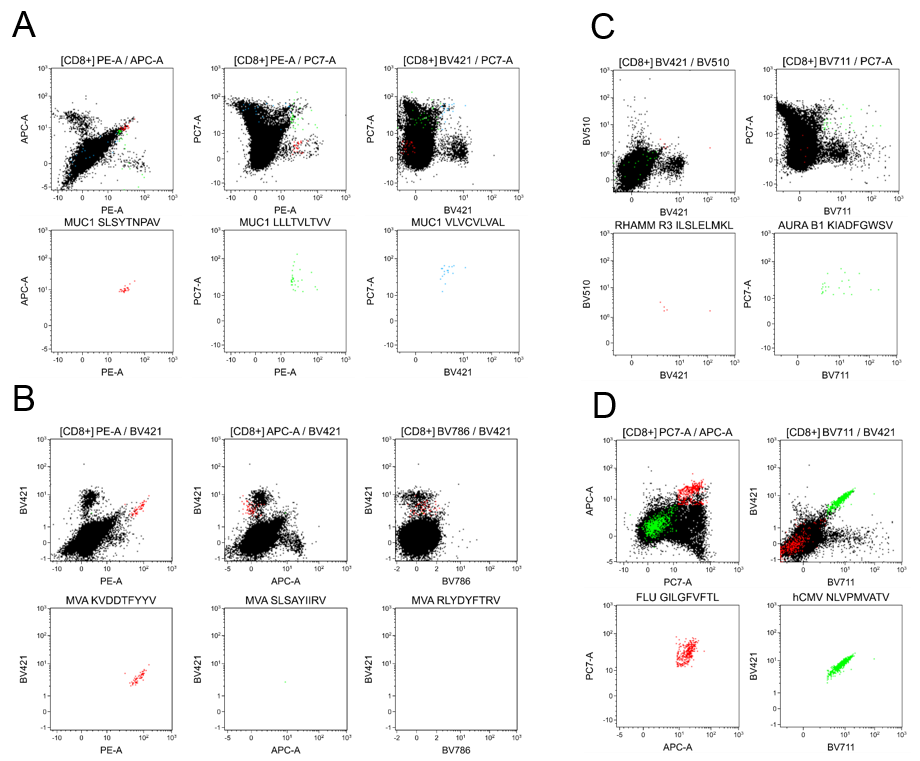


Supplementary figure 4: Same as in supplementary figure 3 for patient 201_00001 from the TG4010 arm. In C, HLA-A02*01-restricted epitopes of MAGEA3 (FLWGPRALV) and PRAMEP3 (ALYVDSLFFL) are shown.


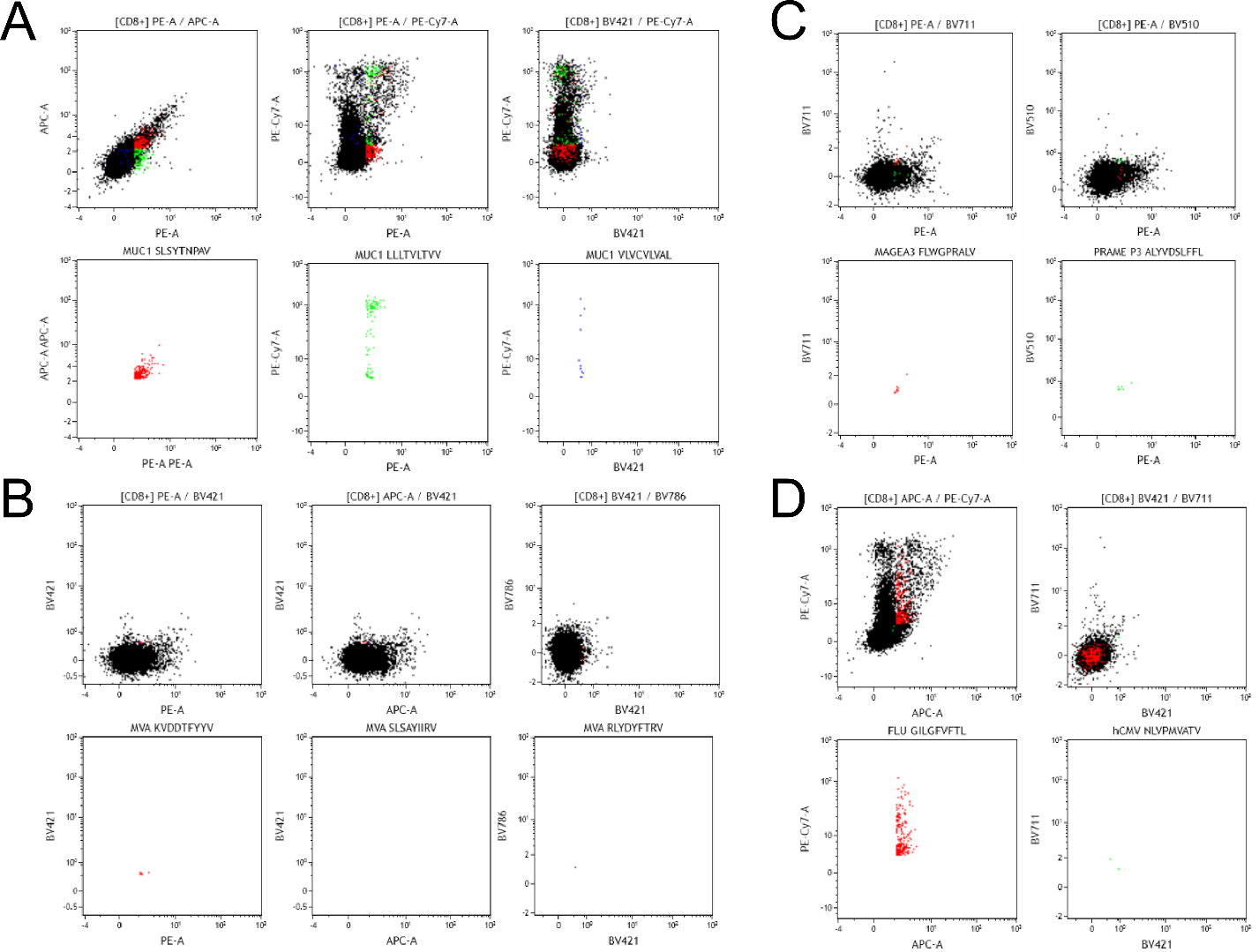


Supplementary Fig. 5. Kaplan-Meier plots of survival in the TG4010 (left panels) and Placebo arm (right panels) stratified on response against Flu (upper panels) and hCMV (lower panels). Patients were stratified based on the response intensity and allocated to the “high” subgroup (blue line, placebo n=19, TG4010 n=20) when above median or “low” group (green line, placebo n=12, TG4010 n=27) when below median. (ns: not significant, Log rank test).

Supplementary Table 1: Viability of the thawed samples used to monitor T cell response. Baseline samples were pooled with C1D1+6h samples to obtain enough cells to measure frequencies of specific CD8+ T cell responses prior to treatment. Accordingly, C3D1, C5D1 and EoT samples were pooled to have enough cells to measure frequencies of specific CD8+ T cell responses after treatment. Some samples were not pooled (italic) when viability was lower than 80% and enough cells were harvested to perform the assay. Otherwise, lower viability samples were also pooled to obtain enough cells. A viability marker was included in the staining to eliminate dead cells from the analysis. nd: not done.

| **Patients** | **Treatment Arm** | **Visit** | | **Cell Number** | | **viability %** | |
| --- | --- | --- | --- | --- | --- | --- | --- |
| 0101_00024 | PLACEBO | Baseline | | 4.70E+05 | | 78 | |
|  |  | C1D1+6h | | 2.70E+05 | | 80 | |
|  |  | C3D1 | | 6.10E+06 | | 89 | |
|  |  | C5D1 | | 1.10E+07 | | 94 | |
|  |  | EoT | | 1.70E+07 | | 93 | |
| 0102_00002 | PLACEBO | Baseline | | 4.70E+06 | | 92 | |
|  |  | *C1D1+6h* | | *2.20E+06* | | *<50* | |
|  |  | C3D1 | | 7.10E+06 | | 90 | |
|  |  | C5D1 | | 7.20E+06 | | 90 | |
|  |  | *EoT* | | *6.00E+06* | | *<50* | |
| 0106_00001 | TG4010 | Baseline | | 2.90E+06 | | 84 | |
|  |  | C1D1+6h | | 1.10E+07 | | 88 | |
|  |  | C1D15 | | 3.10E+06 | | 96 | |
|  |  | C3D1 | | 2.20E+07 | | 96 | |
|  |  | EoT | | 5.00E+06 | | 92 | |
| 0102_00006 | TG4010 | Baseline | | 1.15E+07 | | 87 | |
|  |  | C1D1+6h | | 3.80E+06 | | 84 | |
|  |  | C1D15 | | 7.10E+06 | | 86 | |
|  |  | C3D1 | | 1.84E+07 | | 97 | |
|  |  | EoT | | 2.20E+06 | | 82 | |
| 0108_00005 | TG4010 | Baseline | | 6.70E+06 | | 93 | |
|  |  | C1D1+6h | | 5.20E+06 | | 95 | |
|  |  | C3D1 | | 5.70E+06 | | 96 | |
|  |  | EoT | | 4.60E+05 | | 88 | |
| 0108_00022 | PLACEBO | Baseline | | 6.40E+06 | | 89 | |
|  |  | *C1D1+6h* | | *nd* | | *<50* | |
|  |  | C3D1 | | 6.50E+06 | | 80 | |
|  |  | C5D1 | | 1.60E+07 | | 86 | |
|  |  | EoT | | 5.70E+06 | | 91 | |
| 0109_00008 | PLACEBO | Baseline | | 7.00E+06 | | 95 | |
|  |  | C1D1+6h | | 8.00E+06 | | 91 | |
|  |  | *C3D1* | | *nd* | | *<50* | |
|  |  | C5D1 | 5.80E+06 | | 97 | |  |
|  |  | EoT | 2.23E+07 | | 88 | |  |
| 0109_00009 | TG4010 | Baseline | 5.00E+06 | | 95 | |  |
|  |  | C1D1+6h | 2.30E+06 | | 95 | |  |
|  |  | C3D1 | 4.00E+06 | | 95 | |  |
|  |  | C5D1 | 3.00E+06 | | 84 | |  |
|  |  | *EoT* | | *nd* | | *<50* | |
| 0110_00001 | TG4010 | Baseline | | 2.30E+07 | | 96 | |
|  |  | C1D1+6h | | 5.30E+06 | | 94 | |
|  |  | C3D1 | | 1.02E+07 | | 97 | |
|  |  | EoT | | 8.10E+06 | | 98 | |
| 0115_00001 | TG4010 | Baseline | | 3.00E+06 | | 98 | |
|  |  | C1D1+6h | | 3.00E+06 | | 96 | |
|  |  | C3D1 | | 5.00E+06 | | 95 | |
|  |  | C5D1 | | 5.00E+06 | | 91 | |
|  |  | EoT | | 6.10E+06 | | 97 | |
| 0115_00007 | PLACEBO | Baseline | | 1.40E+07 | | 96 | |
|  |  | C1D1+6h | | 7.20E+06 | | 80 | |
|  |  | C3D1 | | 1.30E+07 | | 94 | |
|  |  | C5D1 | | 5.30E+06 | | 99 | |
|  |  | EoT | | 1.70E+06 | | 96 | |
| 0116_00001 | TG4010 | Baseline | | 9.70E+06 | | 88 | |
|  |  | C1D1+6h | | 3.90E+06 | | 91 | |
|  |  | C3D1 | | 7.10E+06 | | 96 | |
|  |  | EoT | | 9.50E+06 | | 97 | |
| 0116_00004 | TG4010 | Baseline | | 4.30E+06 | | 89 | |
|  |  | C1D1+6h | | 1.12E+07 | | 82 | |
|  |  | C3D1 | | 5.90E+06 | | 84 | |
|  |  | C5D1 | | 1.20E+07 | | 77 | |
|  |  | *EoT* | | *nd* | | *<50* | |
| 0201_00001 | TG4010 | Baseline | | 7.70E+06 | | 96 | |
|  |  | C1D1+6h | | 2.80E+06 | | 78 | |
|  |  | *C3D1* | | *3.10E+07* | | *74* | |
|  |  | *C5D1* | | *1.70E+07* | | *70* | |
|  |  | EoT | | 1.11E+07 | | 96 | |
| 0203_00016 | TG4010 | Baseline | | 1.30E+07 | | 95 | |
|  |  | C1D1+6h | | 5.30E+06 | | 87 | |
|  |  | C3D1 | | 1.50E+07 | | 80 | |
|  |  | C5D1 | | 1.14E+07 | | 84 | |
|  |  | EoT | | 2.20E+07 | | 83 | |
| 0203_00018 | TG4010 | Baseline | | 1.65E+07 | | 92 | |
|  |  | C1D1+6h | | 5.30E+06 | | 78 | |
|  |  | *C3D1* | | *1.30E+07* | | *58* | |
|  |  | C5D1 | | 1.10E+07 | | 87 | |
|  |  | *EoT* | | *nd* | | *<50* | |
| 0203_00024 | TG4010 | Baseline | | 1.14E+07 | | 88 | |
|  |  | C1D1+6h | | 3.75E+06 | | 89 | |
|  |  | C3D1 | | 8.00E+06 | | 81 | |
|  |  | C5D1 | | 8.35E+06 | | 89 | |
| 0205_00004 | PLACEBO | Baseline | | 1.00E+07 | | 93 | |
|  |  | C1D1+6h | | 3.70E+06 | | 93 | |
|  |  | C3D1 | | 1.84E+07 | | 89 | |
|  |  | EoT | | 3.00E+07 | | 86 | |
| 0205_00006 | TG4010 | Baseline | | 9.40E+06 | | 96 | |
|  |  | *C1D1+6h* | | *1.75E+07* | | *<50* | |
|  |  | C3D1 | | 2.30E+07 | | 72 | |
|  |  | *EoT* | | *1.24E+07* | | *44* | |
| 0205_00007 | PLACEBO | Baseline | | 1.60E+07 | | 74 | |
|  |  | *C1D1+6h* | | *8.70E+05* | | *54* | |
|  |  | C3D1 | | 2.00E+06 | | 88 | |
|  |  | C5D1 | | 7.00E+06 | | 84 | |
|  |  | EoT | | 7.00E+06 | | 92 | |
| 0601_00015 | TG4010 | Baseline | | 4.00E+06 | | 92 | |
|  |  | C1D1+6h | | 5.23E+06 | | 85 | |
|  |  | C3D1 | | 7.40E+06 | | 82 | |
|  |  | C5D1 | | 5.43E+06 | | 87 | |
|  |  | EoT | | 6.70E+06 | | 91 | |
| 0701_00007 | TG4010 | Baseline | | 5.70E+06 | | 89 | |
|  |  | C1D1+6h | | 7.10E+06 | | 91 | |
|  |  | C3D1 | | 5.00E+06 | | 87 | |
|  |  | C5D1 | | 2.80E+06 | | 84 | |
| 0703_00007 |  | Baseline | | 9.00E+06 | | 94 | |
|  |  | C1D1+6h | | 1.70E+06 | | 94 | |
|  |  | *C3D1* | | *1.10E+07* | | *70* | |
|  |  | C5D1 | | 1.70E+06 | | 90 | |
|  |  | *EoT* | | *3.00E+07* | | *66* | |
| 0703_00009 | PLACEBO | *Baseline* | | *9.23E+06* | | *48* | |
|  |  | C1D1+6h | | 4.40E+06 | | 78 | |
|  |  | C3D1 | | 6.10E+06 | | 87 | |
|  |  | C5D1 | | 1.31E+07 | | 83 | |
|  |  | *EoT* | | *1.68E+07* | | *58* | |
| 0703_00011 | PLACEBO | Baseline | | 6.00E+06 | | 90 | |
|  |  | C1D1+6h | | 5.00E+05 | | 86 | |
|  |  | *C3D1* | | *4.60E+05* | | *23* | |
|  |  | C5D1 | | 9.00E+05 | | 92 | |
|  |  | EoT | | 5.00E+06 | | 97 | |
| 0704_00011 | PLACEBO | *Baseline* | | *1.20E+07* | | *59* | |
|  |  | C1D1+6h | | 5.20E+06 | | 87 | |
|  |  | C3D1 | | 1.23E+07 | | 89 | |
|  |  | C5D1 | | 1.22E+07 | | 79 | |
|  |  | EoT | | 1.80E+07 | | 81 | |
| 2002_00005 | PLACEBO | *Baseline* | | *nd* | | *44* | |
|  |  | C1D1+6h | | 1.07E+07 | | 72 | |
|  |  | C3D1 | | 6.65E+06 | | 73 | |
|  |  | C5D1 | | 7.85E+06 | | 87 | |
|  |  | EoT | | 8.20E+06 | | 72 | |
| 2003_00002 | TG4010 | Baseline | | 4.00E+06 | | 76 | |
|  |  | C1D1+6h | | 2.00E+06 | | 85 | |
|  |  | C3D1 | | 1.30E+07 | | 93 | |
|  |  | EoT | | 9.00E+06 | | 96 | |
| 2007_00006 | TG4010 | Baseline | | 5.10E+06 | | 88 | |
|  |  | C1D1+6h | | 3.00E+06 | | 95 | |
|  |  | C3D1 | | 4.00E+06 | | 95 | |
|  |  | EoT | | 8.00E+06 | | 95 | |
| 2007_00007 | TG4010 | Baseline | | 1.30E+07 | | 83 | |
|  |  | C1D1+6h | | 1.20E+07 | | 86 | |
|  |  | C3D1 | | 1.60E+07 | | 90 | |
|  |  | C5D1 | | 1.50E+07 | | 94 | |
|  |  | EoT | | 1.70E+07 | | 95 | |
| 2007_00009 | TG4010 | Baseline | | 1.50E+07 | | 97 | |
|  |  | C1D1+6h | | 4.00E+06 | | 96 | |
|  |  | C3D1 | | 8.00E+07 | | 88 | |
|  |  | C5D1 | | 3.00E+07 | | 84 | |
|  |  | EoT | | 2.30E+07 | | 94 | |
| 2301_00006 | TG4010 | Baseline | | 7.00E+06 | | 98 | |
|  |  | C1D1+6h | | 7.00E+06 | | 99 | |
|  |  | C3D1 | | 9.00E+06 | | 94 | |
|  |  | C5D1 | | 2.00E+07 | | 97 | |
|  |  | EoT | | 4.00E+07 | | 95 | |
| 2301_00012 | TG4010 | Baseline | | 3.20E+06 | | 99 | |
|  |  | C1D1+6h | | 1.30E+07 | | 93 | |
|  |  | C3D1 | | 8.20E+06 | | 86 | |
|  |  | C5D1 | | 1.70E+07 | | 82 | |
|  |  | EoT | | 4.20E+06 | | 87 | |
| 2302_00008 | PLACEBO | Baseline | | 2.90E+06 | | 52 | |
|  |  | C1D1+6h | | 8.70E+06 | | 43 | |
|  |  | C3D1 | | 3.50E+07 | | 29 | |
|  |  | EoT | | 8.10E+06 | | 63 | |
| 2305_00003 | TG4010 | Baseline | | 6.66E+06 | | 24 | |
|  |  | C1D1+6h | | 5.50E+06 | | 29 | |
|  |  | C3D1 | | 1.29E+07 | | 71 | |
|  |  | EoT | | 1.03E+07 | | 66 | |
| 2305_00014 | TG4010 | Baseline | | 8.00E+06 | | 85 | |
|  |  | C1D1+6h | | 4.00E+06 | | 57 | |
|  |  | C3D1 | | 7.00E+06 | | 76 | |
|  |  | C5D1 | | 1.50E+06 | | 64 | |
|  |  | EoT | | 1.00E+07 | | 78 | |
| 2305_00022 | TG4010 | Baseline | | 2.00E+07 | | 88 | |
|  |  | *C1D1+6h* | | *2.00E+07* | | *70* | |
|  |  | C3D1 | | 2.50E+07 | | 85 | |
|  |  | *C5D1* | | *2.60E+06* | | *72* | |
|  |  | EoT | | 6.00E+06 | | 87 | |
| 2305_00023 | TG4010 | Baseline | | 7.00E+06 | | 81 | |
|  |  | C1D1+6h | | 5.00E+06 | | 70 | |
|  |  | C3D1 | | 1.50E+07 | | 86 | |
|  |  | EoT | | 9.50E+06 | | 82 | |
| 2306_00001 | PLACEBO | Baseline | | 5.20E+07 | | 70 | |
|  |  | C1D1+6h | | 7.60E+07 | | 69 | |
|  |  | C3D1 | | 2.30E+07 | | 85 | |
|  |  | *EoT* | | *1.40E+06* | | *68* | |
| 2310_00005 | PLACEBO | Baseline | | 1.10E+07 | | 97 | |
|  |  | C1D1+6h | | 3.70E+07 | | 99 | |
|  |  | C3D1 | | 1.00E+08 | | 95 | |
|  |  | EoT | | 8.60E+07 | | 94 | |
| 4003_00001 | PLACEBO | Baseline | | 2.30E+06 | | <50 | |
|  |  | C1D1+6h | | 3.00E+06 | | 78 | |
|  |  | C3D1 | | 3.80E+06 | | 66 | |
|  |  | C5D1 | | 2.80E+06 | | 75 | |
| 4003_00003 | TG4010 | Baseline | | 5.00E+06 | | <50 | |
|  |  | C1D1+6h | | 2.50E+07 | | 76 | |
|  |  | C3D1 | | 1.30E+07 | | 58 | |
|  |  | C5D1 | | 1.30E+07 | | 76 | |
|  |  | EoT | | 1.60E+07 | | 82 | |
| 4003_00008 | TG4010 | Baseline | | 3.70E+06 | | 75 | |
|  |  | C1D1+6h | | 5.50E+05 | | 72 | |
|  |  | C3D1 | | 2.00E+06 | | 69 | |
|  |  | EoT | | 4.50E+06 | | 64 | |
| 4003_00020 | TG4010 | Baseline | | 1.80E+07 | | 95 | |
|  |  | C1D1+6h | | 1.50E+06 | | 90 | |
|  |  | C3D1 | | 1.10E+07 | | 57 | |
|  |  | C5D1 | | 6.00E+06 | | 63 | |
|  |  | EoT | | 1.10E+07 | | 49 | |
| 4003_00016 | TG4010 | Baseline | | 8.00E+06 | | 71 | |
|  |  | C1D1+6h | | 9.00E+06 | | 78 | |
|  |  | C3D1 | | 1.20E+07 | | 73 | |
|  |  | EoT | | 4.80E+06 | | 71 | |
| 0101_00013 | TG4010 | Baseline | | 3.20E+06 | | 74 | |
|  |  | C1D1+6h | | 1.20E+07 | | 63 | |
|  |  | C3D1 | | 4.30E+07 | | 60 | |
|  |  | EoT | | 1.40E+07 | | 85 | |
| 0108_00007 | TG4010 | Baseline | | 1.00E+07 | | 85 | |
|  |  | C1D1+6h | | 8.00E+06 | | 86 | |
|  |  | C3D1 | | 3.00E+06 | | 88 | |
|  |  | EoT | | 1.30E+07 | | 82 | |
| 0101_00019 | TG4010 | Baseline | | 2.60E+06 | | 76 | |
|  |  | C1D1+6h | | 1.10E+07 | | 67 | |
|  |  | C3D1 | | 3.60E+07 | | 79 | |
|  |  | EoT | | 1.00E+07 | | 73 | |
| 2307_00001 | TG4010 | Baseline | | 2.60E+07 | | 72 | |
|  |  | C1D1+6h | | 1.70E+07 | | 53 | |
|  |  | C3D1 | | 9.00E+06 | | 89 | |
|  |  | C5D1 | | 1.60E+07 | | 60 | |
|  |  | EoT | | 3.80E+07 | | 81 | |
| 0101_00025 | TG4010 | Baseline | | 1.00E+07 | | 82 | |
|  |  | C1D1+6h | | 4.85E+06 | | 76 | |
|  |  | C3D1 | | 1.50E+07 | | 83 | |
|  |  | EoT | | 6.00E+06 | | 91 | |
| 0102_00020 | PLACEBO | Baseline | | 3.50E+06 | | 82 | |
|  |  | C1D1+6h | | 2.40E+07 | | 50 | |
|  |  | C3D1 | | 6.00E+06 | | 79 | |
|  |  | EoT | | 8.00E+06 | | 80 | |
| 0108_00023 | TG4010 | Baseline | | 1.65E+06 | | 90 | |
|  |  | C1D1+6h | | 1.45E+07 | | 94 | |
|  |  | C3D1 | | 3.00E+07 | | 79 | |
|  |  | EoT | | 1.50E+07 | | 93 | |
| 0111_00003 | PLACEBO | Baseline | | 6.00E+06 | | 83 | |
|  |  | C1D1+6h | | 2.45E+06 | | 60 | |
|  |  | C3D1 | | 5.00E+06 | | 85 | |
|  |  | EoT | | 5.50E+06 | | 65 | |
| 0112_00001 | TG4010 | Baseline | | 2.60E+06 | | 91 | |
|  |  | C1D1+6h | | 5.50E+06 | | 65 | |
|  |  | C3D1 | | 3.80E+06 | | 78 | |
|  |  | C5D1 | | 4.70E+05 | | 81 | |
|  |  | EoT | | 4.30E+06 | | 57 | |
| 0112_00005 | TG4010 | Baseline | | 5.70E+06 | | 87 | |
|  |  | C1D1+6h | | 4.10E+06 | | 78 | |
|  |  | C3D1 | | 2.05E+06 | | 78 | |
|  |  | EoT | | 6.10E+06 | | 66 | |
| 0112_00007 | TG4010 | Baseline | | 1.70E+07 | | 74 | |
|  |  | C1D1+6h | | 4.05E+06 | | 75 | |
|  |  | C3D1 | | 4.20E+06 | | 85 | |
|  |  | EoT | | 3.30E+06 | | 85 | |
| 0113_00001 | PLACEBO | Baseline | | 1.40E+07 | | 86 | |
|  |  | C1D1+6h | | 8.50E+06 | | 74 | |
|  |  | C3D1 | | 6.50E+06 | | 77 | |
|  |  | C5D1 | | 6.00E+06 | | 90 | |
|  |  | EoT | | 3.10E+06 | | 88 | |
| 0115_00003 | TG4010 | Baseline | | 1.74E+06 | | 97 | |
|  |  | C1D1+6h | | 8.74E+05 | | 92 | |
|  |  | C3D1 | | 1.40E+06 | | 85 | |
|  |  | EoT | | 6.72E+05 | | 89 | |
| 0116_00009 | PLACEBO | Baseline | | 1.80E+06 | | 76 | |
|  |  | C3D1 | | 1.80E+06 | | 79 | |
|  |  | EoT | | 4.00E+05 | | 81 | |
| 0201_00003 | TG4010 | Baseline | | 1.50E+06 | | 87 | |
|  |  | C1D1+6h | | 9.00E+05 | | 91 | |
|  |  | C3D1 | | 1.40E+06 | | 87 | |
|  |  | EoT | | 7.10E+05 | | 81 | |
| 0205_00002 | PLACEBO | Baseline | | 4.50E+06 | | 89 | |
|  |  | C1D1+6h | | 6.00E+06 | | 90 | |
|  |  | C3D1 | | 1.50E+07 | | 81 | |
|  |  | C5D1 | | 4.60E+06 | | 85 | |
|  |  | EoT | | 1.10E+07 | | 83 | |
| 2301_00005 | TG4010 | Baseline | | 5.50E+06 | | 89 | |
|  |  | C1D1+6h | | 8.00E+06 | | 77 | |
|  |  | C3D1 | | 1.30E+07 | | 87 | |
|  |  | EoT | | 8.50E+06 | | 67 | |
| 2303_00003 | PLACEBO | Baseline | | 2.80E+07 | | 55 | |
|  |  | C1D1+6h | | 1.30E+07 | | 72 | |
|  |  | C3D1 | | 1.20E+07 | | 89 | |
|  |  | C5D1 | | 7.30E+06 | | 90 | |
|  |  | EoT | | 1.85E+07 | | 71 | |
| 0106_00002 | TG4010 | Baseline | | 1.80E+07 | | 86 | |
|  |  | C1D1+6h | | 9.00E+06 | | 91 | |
|  |  | EoT | | 7.60E+06 | | 78 | |
| 0108_00010 | PLACEBO | Baseline | | 5.80E+06 | | 83 | |
|  |  | EoT | | 5.80E+06 | | 80 | |
| 0101_00003 | PLACEBO | Baseline | | 1.15E+06 | | 91 | |
|  |  | C1D1+6h | | 5.50E+06 | | 82 | |
|  |  | EoT | | 4.60E+06 | | 89 | |
| 0101_00012 | TG4010 | Baseline | | 9.00E+06 | | 84 | |
|  |  | C1D1+6h | | 4.50E+06 | | 82 | |
|  |  | EoT | | 1.60E+07 | | 73 | |
| 0103_00001 | PLACEBO | Baseline | | 3.60E+06 | | 93 | |
|  |  | C1D1+6h | | 7.60E+07 | | <50 | |
|  |  | EoT | | 1.70E+06 | | 86 | |
| 0108_00006 | PLACEBO | Baseline | | 5.20E+06 | | 94 | |
|  |  | C1D1+6h | | 5.50E+06 | | 87 | |
|  |  | EoT | | 1.10E+06 | | 97 | |
| 0201_00004 |  | Baseline | | 4.50E+06 | | 95 | |
|  |  | C1D1+6h | | 1.40E+07 | | 85 | |
|  |  | EoT | | 5.70E+05 | | 82 | |
| 0203_00017 | PLACEBO | Baseline | | 2.30E+07 | | 77 | |
|  |  | C1D1+6h | | 5.50E+06 | | 93 | |
|  |  | EoT | | 2.00E+05 | | 99 | |
| 2003_00001 | PLACEBO | Baseline | | 1.50E+06 | | 81 | |
|  |  | C1D1+6h | | 7.50E+05 | | 94 | |
|  |  | C3D1 | | 7.00E+05 | | 80 | |
|  |  | C5D1 | | 1.00E+06 | | 94 | |
|  |  | EoT | | 4.00E+05 | | 77 | |
| 2007_00024 | TG4010 | Baseline | | 2.30E+06 | | 97 | |
|  |  | C1D1+6h | | 2.70E+06 | | 96 | |
|  |  | EoT | | 4.00E+06 | | 88 | |
| 2301_00011 |  | Baseline | | 1.80E+06 | | 79 | |
|  |  | C1D1+6h | | 1.60E+06 | | 86 | |
|  |  | EoT | | 8.60E+06 | | 83 | |
| 6106_00002 | PLACEBO | Baseline | | 2.50E+06 | | 54 | |
|  |  | C1D1+6h | | 1.20E+06 | | 70 | |
|  |  | EoT | | 5.50E+06 | | 62 | |
| 0108_00028 | PLACEBO | Baseline | | 4.30E+06 | | 92 | |
|  |  | C1D1+6h | | 1.40E+07 | | 74 | |
|  |  | EoT | | 9.00E+06 | | 92 | |
| 0201_00007 | TG4010 | Baseline | | 3.10E+06 | | 89 | |
|  |  | C1D1+6h | | 2.30E+06 | | 79 | |
|  |  | EoT | | 9.20E+06 | | 87 | |
| 0601_00016 | TG4010 | Baseline | | 1.25E+07 | | 89 | |
|  |  | EoT | | 9.20E+06 | | 88 | |
| 2305_00007 | PLACEBO | Baseline | | 1.34E+07 | | 69 | |
|  |  | C1D1+6h | | 2.26E+07 | | 57 | |
|  |  | EoT | | 3.70E+05 | | 73 | |
| 2305_00015 | PLACEBO | Baseline | | 1.03E+07 | | 57 | |
|  |  | C1D1+6h | | 1.97E+06 | | 80 | |
|  |  | EoT | | 1.80E+06 | | 85 | |
| 2007_00001 |  | Baseline | | 3.40E+07 | | 77 | |
|  |  | C1D1+6h | | 1.70E+07 | | 88 | |
|  |  | C3D1 | | 1.10E+07 | | 89 | |
|  |  | EoT | | 2.00E+07 | | 74 | |
| 0111_00004 | TG4010 | Baseline | | 6.30E+06 | | 85 | |
|  |  | C1D1+6h | | 8.10E+06 | | 81 | |
|  |  | EoT | | 1.30E+08 | | <50 | |

Supplementary Table 2: Epitopes used for the measurement of T-cell response. Antigens used for the measurement of T-cell response, corresponding epitope sequences and fluorophores used for staining.

| **MUC1 epitopes** | | |
| --- | --- | --- |
| **Fluorophores** | **Epitope** | **Sequence** |
| PE/APC | MUC1 1240-1248 | SLSYTNPAV |
| PE/PC7 | MUC1 13-21 | LLLTVLTVV |
| BV421/PC7 | MUC1 1165-1173 | VLVCVLVAL |
| **MVA antigen epitopes** | | |
| **Fluorophores** | **Epitope** | **Sequence** |
| APC/BV421 | MVA 184-192 | SLSAYIIRV |
| PE/BV421 | MVA 74-82 | KVDDTFYYV |
| BV421/BV786 | MVA 211-219 | RLYDYFTRV |
| **Tumor associated antigen epitopes** | | |
| **Fluorophores** | **Antigen** | **Sequence** |
| PE/BV711 | MAGE 3 | FLWGPRALV |
| PE/BV510 | PRAME P3 | ALYVDSLFFL |
| BV421/BV510 | RHAMMR3 | ILSLELMKL |
| BV510/BV786 | hTERT | ILAKFLHWL |
| BV510/BV711 | G250 CA-1X | QLLLSLLLL |
| BV510/BV605 | Her2/neu | RLLQETELV |
| BV711/BV786 | SURVIVIN | ELTLGEFLKL |
| PE/BV605 | MAGE 3 | KVAELVHFL |
| BV421/BV605 | WT1 P | RMFPNAPYL |
| BV711/PC7 | AURA B1 | KIADFGWSV |
| BV605/BV786 | AURA A1 | TLCGTLDYL |
| BV605/BV711 | EGFR G719S | VLSSGAFGTV |
| APC/BV605 | CDN2A A57V | VMMMGSVRV |
| BV510/PC7 | ATM T2666A | NLEDVVVPA |
| APC/BV711 | TP53 K132N | ALNNMFCQL |
| **Control viral antigen epitopes** | | |
| **Fluorophores** | **Antigen** | **Sequence** |
| *APC/PC7* | *Flu Matrix 58-66* | *GILGFVFTL* |
| *BV421/BV711* | *hCMV 495-503* | *NLVPMVATV* |

Supplementary Table 3: Baseline characteristics for patients with response against 0 or 1 MUC1 epitopes (Low diversity) and 2 or 3 MUC1 epitopes (High diversity). (non-parametric Wilcoxon-Mann-Whitney test and for categorical parameters the Fisher exact test)

| **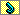Baseline characteristics** | **Low Diversity MUC1 response** | **High Diversity MUC1 response** | **Overall** | ***p*-value** |
| --- | --- | --- | --- | --- |
| N | 25 | 53 | 78 |  |
| Gender |  |  |  | 1.00 |
| Male | 17 (68.0%) | 36 (67.9%) | 53 (67.9%) |  |
| Female | 8 (32.0%) | 17 (32.1%) | 25 (32.1%) |  |
| Age (years) |  |  |  | 0.82 |
| Mean (SD) | 59.8 (10.01) | 60.4 (7.98) | 60.2 (8.62) |  |
| Median | 62.0 | 61.0 | 61.0 |  |
| Min - Max | 36.0 - 77.0 | 38.0 - 77.0 | 36.0 - 77.0 |  |
| Q1 - Q3 | 54.0 - 65.0 | 56.0 - 66.0 | 55.0 - 66.0 |  |
| Body Mass Index (kg/m²) |  |  |  | 0.64 |
| Mean (SD) | 24.3 (4.09) | 25.2 (5.11) | 24.9 (4.79) |  |
| Median | 24.8 | 24.9 | 24.8 |  |
| Min - Max | 16.4 - 35.2 | 16.8 - 40.8 | 16.4 - 40.8 |  |
| Q1 - Q3 | 21.7 - 26.4 | 22.0 - 27.8 | 21.9 - 26.8 |  |
| Stage at initial diagnosis |  |  |  | 0.86 |
| Stage IA | 0 (0.0%) | 1 (1.9%) | 1 (1.3%) |  |
| Stage IIA | 0 (0.0%) | 1 (1.9%) | 1 (1.3%) |  |
| Stage IIB | 0 (0.0%) | 2 (3.8%) | 2 (2.6%) |  |
| Stage IIIA | 0 (0.0%) | 1 (1.9%) | 1 (1.3%) |  |
| Stage IV | 25 (100.0%) | 48 (90.6%) | 73 (93.6%) |  |
| Time since initial diagnosis (months) |  |  |  | 0.97 |
| Mean (SD) | 1.44 (0.651) | 3.27 (7.037) | 2.68 (5.858) |  |
| Median | 1.28 | 1.38 | 1.37 |  |
| Min - Max | 0.56 - 3.55 | 0.33 - 46.88 | 0.33 - 46.88 |  |
| Q1 - Q3 | 1.12 - 1.51 | 0.82 - 2.07 | 0.92 - 1.91 |  |
| Histology |  |  |  | 1.00 |
| Adenocarcinoma | 23 (92.0%) | 45 (84.9%) | 68 (87.2%) |  |
| Squamous Cell Carcinoma | 2 (8.0%) | 6 (11.3%) | 8 (10.3%) |  |
| Large Cell Carcinoma | 0 (0.0%) | 1 (1.9%) | 1 (1.3%) |  |
| Other | 0 (0.0%) | 1 (1.9%) | 1 (1.3%) |  |
| Primary tumor focality |  |  |  | 0.46 |
| Unifocal | 16 (64.0%) | 34 (64.2%) | 50 (64.1%) |  |
| Multifocal | 1 (4.0%) | 0 (0.0%) | 1 (1.3%) |  |
| Unknown | 8 (32.0%) | 19 (35.8%) | 27 (34.6%) |  |
| Performance Status (PS/ECOG) |  |  |  | 0.45 |
| Fully Active | 7 (28.0%) | 21 (39.6%) | 28 (35.9%) |  |
| Restricted In Physically Strenuous Activity | 18 (72.0%) | 32 (60.4%) | 50 (64.1%) |  |

Supplementary Table 4: Baseline characteristics for patients with detected MUC1 response at baseline, with acquired response during treatment or with no response detected after treatment. (non-parametric Wilcoxon-Mann-Whitney test and for categorical parameters the Fisher exact test)

| **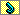Baseline characteristics** | **Baseline MUC1 response** | **Acquired response during treatment** | **No MUC1 response** | **Overall** | ***p*-value** |
| --- | --- | --- | --- | --- | --- |
| N | 41 | 27 | 10 | 78 |  |
| Gender |  |  |  |  | 0.75 |
| Male | 27 (65.9%) | 18 (66.7%) | 8 (80.0%) | 53 (67.9%) |  |
| Female | 14 (34.1%) | 9 (33.3%) | 2 (20.0%) | 25 (32.1%) |  |
| Age (years) |  |  |  |  | 0.38 |
| Mean (SD) | 60.0 (7.31) | 59.8 (8.84) | 62.0 (12.99) | 60.2 (8.62) |  |
| Median | 61.0 | 59.0 | 66.0 | 61.0 |  |
| Min - Max | 44.0 - 77.0 | 38.0 - 77.0 | 36.0 - 77.0 | 36.0 - 77.0 |  |
| Q1 - Q3 | 55.0 - 65.0 | 55.0 - 66.0 | 60.0 - 71.0 | 55.0 - 66.0 |  |
| Body Mass Index (kg/m²) |  |  |  |  | 0.37 |
| Mean (SD) | 24.5 (5.26) | 25.7 (4.73) | 24.7 (2.46) | 24.9 (4.79) |  |
| Median | 24.0 | 25.7 | 24.9 | 24.8 |  |
| Min - Max | 16.4 - 40.8 | 16.8 - 39.2 | 21.0 - 29.1 | 16.4 - 40.8 |  |
| Q1 - Q3 | 21.2 - 26.4 | 22.5 - 27.8 | 23.2 - 26.6 | 21.9 - 26.8 |  |
| Stage at initial diagnosis |  |  |  |  | 0.46 |
| Stage IA | 0 (0.0%) | 1 (3.7%) | 0 (0.0%) | 1 (1.3%) |  |
| Stage IIA | 0 (0.0%) | 1 (3.7%) | 0 (0.0%) | 1 (1.3%) |  |
| Stage IIB | 2 (4.9%) | 0 (0.0%) | 0 (0.0%) | 2 (2.6%) |  |
| Stage IIIA | 0 (0.0%) | 1 (3.7%) | 0 (0.0%) | 1 (1.3%) |  |
| Stage IV | 39 (95.1%) | 24 (88.9%) | 10 (100.0%) | 73 (93.6%) |  |
| Time since initial diagnosis (months) |  |  |  |  | 0.52 |
| Mean (SD) | 2.56 (7.143) | 3.33 (4.674) | 1.44 (0.830) | 2.68 (5.858) |  |
| Median | 1.35 | 1.41 | 1.15 | 1.37 |  |
| Min - Max | 0.53 - 46.88 | 0.33 - 17.77 | 0.72 - 3.55 | 0.33 - 46.88 |  |
| Q1 - Q3 | 0.82 - 1.91 | 1.08 - 2.20 | 0.99 - 1.71 | 0.92 - 1.91 |  |
| Histology |  |  |  |  | 0.57 |
| Adenocarcinoma | 34 (82.9%) | 24 (88.9%) | 10 (100.0%) | 68 (87.2%) |  |
| Squamous Cell Carcinoma | 6 (14.6%) | 2 (7.4%) | 0 (0.0%) | 8 (10.3%) |  |
| Large Cell Carcinoma | 1 (2.4%) | 0 (0.0%) | 0 (0.0%) | 1 (1.3%) |  |
| Other | 0 (0.0%) | 1 (3.7%) | 0 (0.0%) | 1 (1.3%) |  |
| Primary tumor focality |  |  |  |  | 0.16 |
| Unifocal | 28 (68.3%) | 15 (55.6%) | 7 (70.0%) | 50 (64.1%) |  |
| Multifocal | 0 (0.0%) | 0 (0.0%) | 1 (10.0%) | 1 (1.3%) |  |
| Unknown | 13 (31.7%) | 12 (44.4%) | 2 (20.0%) | 27 (34.6%) |  |
| Performance Status (PS/ECOG) |  |  |  |  | 0.08 |
| Fully Active | 19 (46.3%) | 8 (29.6%) | 1 (10.0%) | 28 (35.9%) |  |
| Restricted In Physically Strenuous Activity | 22 (53.7%) | 19 (70.4%) | 9 (90.0%) | 50 (64.1%) |  |

Supplementary Table 5: Number of patients with Low and High diversity MUC1 specific response stratified on TrPAL levels as defined by Quoix et al. (2015).

|  |  | |  | **TrPAL levels** | |
| --- | --- | --- | --- | --- | --- |
|  | | **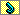** | | **Low** | **Elevated** |
| **Number of patients** |  | | Low diversity MUC1 response | 27 | 15 |
|  |  | | High diversity MUC1 response | 26 | 10 |

Supplementary Table 6: Number of patients with high and low diversity MUC1 response in each treatment arm according to the concomitant chemotherapy regimen.

|  | **MUC1 response** | | | |
| --- | --- | --- | --- | --- |
|  | **High Diversity** | | **Low Diversity** | |
|  | **TG4010** | **Placebo** | **TG4010** | **Placebo** |
| **Carboplatin + Paclitaxel** | 2 | 2 | 9 | 7 |
| **Cisplatin-based regimen** | 14 | 7 | 22 | 15 |
| **Total** | 16 | 9 | 31 | 22 |

Supplementary Table 7: Number of patients with high and low diversity MVA response in each treatment arm according to the concomitant chemotherapy regimen.

|  | **MVA response** | | | |
| --- | --- | --- | --- | --- |
|  | **High Diversity** | | **Low Diversity** | |
|  | **TG4010** | **Placebo** | **TG4010** | **Placebo** |
| **Carboplatin + Paclitaxel** | 4 | 1 | 7 | 8 |
| **Cisplatin-based regimen** | 20 | 9 | 16 | 13 |
| **Total** | 24 | 10 | 23 | 21 |

Supplementary Table 8: Number of patients in groups of MUC1 expression levels with low or high diversity MUC1 specific T-cell response.

|  |  | **Percentage of tumor cells with MUC1 expression** | | | | |
| --- | --- | --- | --- | --- | --- | --- |
| **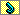** | | **<70%** | **70%-79%** | **80%-89%** | **90%-99%** | ***>99%*** |
| **Number of patients** | Low diversity MUC1 response | 0 | 5 | 4 | 10 | 34 |
|  | High diversity MUC1 response | 0 | 1 | 2 | 4 | 18 |
