# Supplementary material for: Viral based vaccine TG4010 induces broadening of specific immune response and improves outcome in advanced NSCLC
Source: J Immunother Cancer. 2017 Sep 19;5:70. doi: 10.1186/s40425-017-0274-x (PMC5604422; doi:10.1186/s40425-017-0274-x)

Validation of multiparametric tetramer staining

Principle of the validation

While the detection of serological response is well described and objects of numerous guidance, much less information is available on validation of methodology for the detection of cellular immunologic response responses. A recently developed approach uses tetrameric complexes of HLA molecules to stain antigen-specific T cells in FACS analysis. Tetramer staining presents advantages over traditional ELISPOT assays as it does not require a cell stimulation period likely to exert a selective pressure on T-cell subpopulations. However, interpretation of tetramer staining in the setting of cancer immunotherapeutic products presents a challenge because of the low number of cells involved in individual epitope response that can be detected in the peripheral circulation.

The validation of the method intends to support interpretation of the results. The main objective of the validation is to ensure that positive signal can be ascertained from background signal in spite of low event rates. While number of replicates used in the validation are lower (6) compared to number of replicates usually recommended in assay validation guidelines (30 or more), it is to be noted that cytometric measurements are the results of an assessment made in population of several thousands of cells. It was shown that measurements of 5 individual samples are sufficient to derive significant statistics on the method performance.

Validation samples

Samples used during validation are described as follow:

- $V_{HV}1$, $V_{HV}2$ $V_{HV}3$, $V_{HV}4$, $V_{HV}5$, to $V_{HV}6$: Are PBMC samples obtained from qualified blood donors. These subjects are presumably not affected by neoplastic disorders and therefore are unlikely to exhibit positive T-response against cancer associated antigens. Conversely, these samples may however exhibit response to common antigens flu and hCMV directed epitopes..
- V_{FLU+} and V_{FLU-} are used to designate a selected sample showing positive and negative reaction respectively against the flu specific epitope,
- V_{CMV+} and V_{CMV-} are used to designate a selected sample showing respectively a positive and a negative response against a hCMV specific epitope.
- HV_a is a healthy volunteer sample selected for having detectable events for a maximum of different epitope tested.

Validation parameters

Intermediate precision

Intermediate precision CV_{inter} of the assay was calculated using measurements of one given sample across 6 assay runs by two different operators. Intermediate precision is expressed for each tetramer as the coefficient of variation (CV) observed for a measurable event rate. For each combination of peptides (*i,j*):

$$CV_{inter}^{(i,j)} = \frac{\sigma_{HVa}^{(i,j)}}{\mu_{HVa}^{(i,j)}}$$

Intersubject variability

Coefficient of variation $CV_{IS}^{(i,j)}$ associated with T-cells reaction of 6 individuals against an influenza derived epitope will serve to estimate the expectable variation associated with T-cell responses.

$$CV_{IS}^{(i,j)} = \frac{\sigma_{IS}^{(i,j)}}{\mu_{IS}^{(i,j)}}$$

Limit of Blank and Limit of Detection

For each combination of tetramers, The limit of blank is defined as the highest apparent event rate expected to be found when replicates of a sample containing no analyte are tested[3].
In order to determine the LoB for combinations of color (*k,l*) associated with cancer neo-epitopes, event rates observed in 5 replicate measurements of sample HV*a* will be used. LoB for color combination related to neo-epitopes (*k,l*) will be estimated as:

$$LoB_{(k,l)} = \mu_{HV_a}^{(k,l)} + 1.645(\sigma_{HVa}^{(k,l)})$$

Where SD is the intermediate precision of the assay. A value above the LoB level will have a probability of <0.05 to be non-different of background noise.
The LoD will be calculated using the following estimator:

$$LoD = LoB + 1.645(\sigma_{HVa}^{(k,l)})$$

Linearity

Early experience with the assay revealed that T-cell immune-reactivity against hCMV is highly variable across the population. Some subjects may exhibit high level of T-cell activation against hCMV related epitope, while others does not show detectable hCMV reacting T-cells. This may be due to the significant prevalence of subclinical hCMV infection in the general population.
This has allowed identification of both highly positive subjects (up to 1% of anti-hCMV $CD8^+$ cells) and negative subjects. These samples could be used to establish linearity of the method by spiking negative sample with different CMV reacting cells in order to obtain different levels of event rates. Samples were prepared with nominal event rates of 10^{-2} to 10^{-4}.
Data are plotted on a scatterplot and linearity is assumed if the event rate measurement does not deviate of more than 1.96 from the expected value.

Donor 4, negative for a response against CMV, $CD8^+$ T cells (100 µL) were spiked with 100 µL of a CMV positive $CD8^+$ T cells at different dilution (10x, 20x, 50x, 100x, 1000x or Blank sample). Two independent assays were performed.

Linearity of this assay will be represented as:

% of Tet CMV^+ observed by flow cytometry versus % Expected values

Validation results

Intermediate precision

Table 1: Intermediate precision of the method, limit of blank and limit of detection of the method. From 6 independent measurements on the same donor by 2 different operators, and for each combinatorial encoding tested, a mean, standard deviation, intermediate precision (%CV), limit of blank (LoB) and limit of detection (LoD were calculated.

Fluorophores	Antigens	Mean (frequency positive cells)	SD	Intermediate precision (%CV)	LoB	LoD
APC/BV421	MVA	0.0026	0.00291	114	0.0073	0.0121
APC/BV605	CDN2A mutation A57V	0.0027	0.00223	82	0.0064	0.0100
APC/BV711	TP53 mutation K132N	0.0032	0.00279	87	0.0078	0.0124
BV421/BV510	RHAMM-R3	0.0055	0.00408	74	0.0122	0.0189
BV421/BV605	WT1	0.0033	0.00193	59	0.0064	0.0096
BV421/BV786	MVA	0.0013	0.00164	130	0.0040	0.0067
BV421/PC7	MUC1	0.0013	0.00117	89	0.0032	0.0052
BV510/BV605	HER2	0.0047	0.00313	67	0.0098	0.0149
BV510/BV711	G250	0.0054	0.00431	80	0.0125	0.0196
BV510/BV786	Htert	0.0008	0.00071	95	0.0019	0.0031
BV510/PC7	ATM mutation T2666A	0.0016	0.00124	78	0.0036	0.0057
BV605/BV711	EGFR mutation G719S	0.0199	0.01355	68	0.0422	0.0645
BV605/BV786	AURA-A1	0.0018	0.00145	82	0.0041	0.0065
BV711/BV786	SURVIVIN	0.0044	0.00266	61	0.0087	0.0131
BV711/PC7	AURA-B1	0.0069	0.00538	78	0.0157	0.0246
PE/APC	MUC1	0.0197	0.01811	92	0.0495	0.0793
PE/BV421	MVA	0.0042	0.00233	56	0.0080	0.0119
PE/BV510	PRAME-P3	0.0008	0.00080	95	0.0022	0.0035
PE/BV605	MAGE-A3	0.0030	0.00267	90	0.0074	0.0118
PE/BV711	MAGE-A3	0.0046	0.00470	102	0.0123	0.0201
PE/PC7	MUC1	0.0058	0.00270	47	0.0102	0.0147

Intersubject variability

Table 2: Intersubject variability of the method. From 6 independent measurements on 6 different healthy donors a mean, standard deviation and intermediate precision (%CV) were calculated, so as to evaluate the expectable variation associated with T-cell responses

Fluorophores	Antigens	Mean (frequency positive cells)	SD	Intermediate precision (%CV)
APC/PC7	FLU	0.1143	0.204	179
BV421/BV711	hCMV	0.3654	0.592	162

Linearity

Figure 1: Linearity of the method. Linearity was established in 2 independent experiments, by spiking negative sample with different CMV reacting cells in order to obtain different levels of event rates. Samples were prepared with nominal event rates of 10^{-2} to 10^{-4}. The linearity of the CMV Tet^{+} $CD8^{+}$ T cells population was determined by regression analysis with an acceptance criteria of R^2 >0.8.

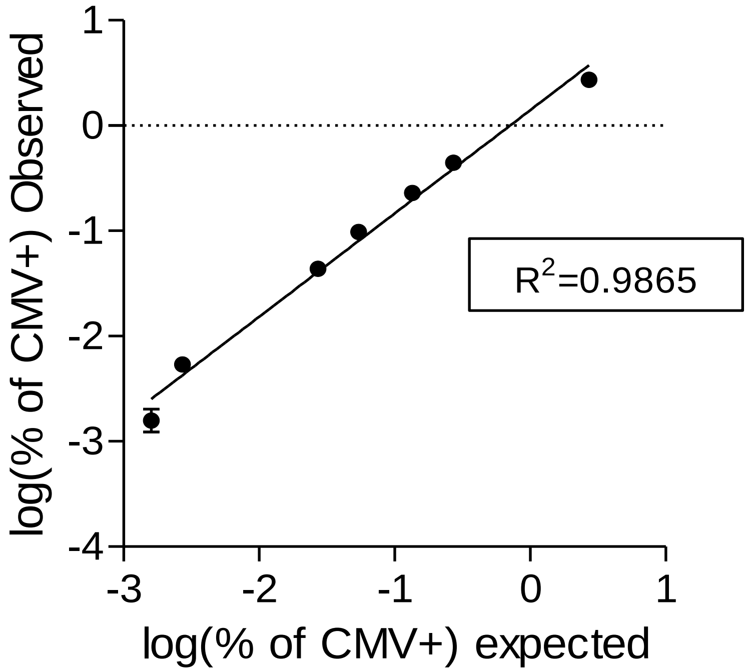

Supplement: Supplementary file 2 — Analytical validation summary reporting analytical performance of the combinatorial tetramer staining assay. (PDF 263 kb) [file 40425_2017_274_MOESM2_ESM.pdf]
