# Supplementary material for: Viral based vaccine TG4010 induces broadening of specific immune response and improves outcome in advanced NSCLC
Source: J Immunother Cancer. 2017 Sep 19;5:70. doi: 10.1186/s40425-017-0274-x (PMC5604422; doi:10.1186/s40425-017-0274-x)
Supplement: Supplementary file 3 — List of ethics committees having reviewed and approved the study protocol. (DOCX 14 kb) [file 40425_2017_274_MOESM3_ESM.docx]

| TG4010.14/TIME - List of Ethics committees | |
| --- | --- |
| Belgium | Central EC:  Comité d’éthique hospitalo-facultaire universitaire de Liège  CHU du Start Tilman B35  4000 Liège  E-mail : [ethique@chu.ulg.ac.be](mailto:ethique@chu.ulg.ac.be) |
| France | Comité de Protection des Personnes Est IV  1, place de l’Hôpital  67091 Strasbourg Cedex |
| Germany | Central EC :  Mediz. Ethikkommission II  Haus 42, Ebene 3  Theodor-Kutzer-Ufer 1-3  68167 Mannheim  E-Mail: [ethikkommission-II@medma.uni-heidelberg.de](mailto:ethikkommission-II@medma.uni-heidelberg.de) |
| Hungary | Central EC:  Egészségügyi Tudományos Tanács  Arany János u. 6-8.  1051 Budapest,  Post mail : 1054 Budapest, Alkotmány u. 25  E-mail: [attilane.gombos@emmi.gov.hu](mailto:attilane.gombos@emmi.gov.hu) |
| Italy | Central EC :  Comitato Etico delle Aziende Sanitarie della Regione Umbria  Via della Rivoluzione, 16  06070 Ellera di Corciano  E-Mail : [segreteria@ceasumbria.it](mailto:segreteria@ceasumbria.it) |
| Poland | Central EC :  Komisja bioetyczna przy oil w szczecinie  Siedziba OIL w Szczecinie pok. nr 6 - II piętro  71332 Szczecin  E-Mail : [bioetyka@oil.szczecin.pl](mailto:bioetyka@oil.szczecin.pl) |
| Spain | Central EC :  Hospital General Universitario Gregorio Marañón  Comité ético de investigacion clinic – Area 1  C/ Dr. Esquerdo 46,  Pabellón de Gobierno,  Planta baja,  28007 Madrid  E-Mail : [ceic.hgugm@salud.madrid.org](mailto:ceic.hgugm@salud.madrid.org) |
| UK | At time of TG4010.14:  West London & GTAC Research Ethics Committee  Nottingham REC Centre  The Old Chapel  Royal Standard Place  NG1 6FS  E-Mail : [NRESCommittee.London-WestLondon@nhs.net](mailto:NRESCommittee.London-WestLondon@nhs.net) |
| US | Central IRB [for Beck, Thambi, Einsphar, Vrindavanam, Sharma]:  Western Institutional Review Board® (WIRB®)  3535 Seventh Avenue, SW  Olympia, Washington 98502  E-mail: [Help@wirb.comWIRB](mailto:Help@wirb.comWIRB)  Local IRB  Mary Crowley medical research center  Institutional Review Board  1700 Pacific avenue  Suite 1100  Dallas, Texas 75201  Local IRB  Mayo clinic Institutional Review Board  Mayo Clinic  201 Building, Room 4-60  200 First St. SW  Rochester, MN 55905  Email: [irbservicecenter@mayo.edu](mailto:irbservicecenter@mayo.edu)  Local IRB  Dana Farber Cancer Institute Institutional Review Board  450 Brookline Avenue, BP332A  Boston, MA 02215  Local IRB  Abington memorial hospital Institutional Review Board  1200 Old York Road  Abington, PA 19001  Local IRB  University of Louisville Institutional Review Board  Med Center One, Suite 200  501 E. Broadway  Louisville, Kentucky 40202 |
